# The essential role of leadership training for physicians in the field of trauma

**DOI:** 10.1590/0100-6991e-20233686EDIT01-en

**Published:** 2023-11-23

**Authors:** Paula Ferrada

**Affiliations:** 1 - University of Virginia, Trauma and Acute Care Surgery - Falls Church - VA - Estados Unidos

## EDITORIAL

Leadership in the field of trauma surgery plays a pivotal role in driving a comprehensive and patient-centric healthcare system. In the dynamic and demanding environment of trauma surgery, physicians are not only expected to possess exceptional medical skills but also to demonstrate effective leadership abilities^1^ ^-^ ^2^. Effective leadership training is a crucial component in preparing physicians to handle the complex challenges and multifaceted responsibilities inherent in trauma surgery. This essay aims to highlight the significance of leadership training for physicians in trauma surgery, underlining the commitment required to cultivate these skills and its potential impact on enhancing patient care on a broader scale^3^ ^-^ ^5^.

## Understanding the Nuances of Leadership in Trauma Surgery

Trauma surgery is a fast-paced, high-stress domain where split-second decisions can mean the difference between life and death. Physicians in this field are often required to lead multidisciplinary teams, make critical decisions under pressure, and effectively communicate with various stakeholders, including patients, families, and other medical professionals. Effective leadership in trauma surgery is not just about making decisions; it encompasses the ability to inspire, motivate, and guide teams to deliver optimal patient outcomes in highly challenging situations. Leadership training equips physicians with the necessary tools and strategies to navigate these complexities while fostering a culture of excellence and collaboration within their teams^5^ ^-^ ^10^.

## Commitment: A Prerequisite for Developing Leadership Skills

Becoming a proficient leader in trauma surgery demands a significant commitment to ongoing learning, personal development, and continuous improvement. Leadership is not an inherent trait but a skill that requires nurturing, practice, and refinement over time. Physicians must dedicate themselves to understanding the intricacies of effective communication, conflict resolution, team management, and strategic decision-making. Commitment to honing these skills allows physicians to build trust, establish credibility, and foster a supportive and cohesive environment within their surgical teams, ultimately contributing to enhanced patient care and improved clinical outcomes^1^ ^-^ ^10^.

## Impact of Leadership Training on Patient Care at a Larger Scale

The importance of effective leadership in trauma surgery extends far beyond the operating room. Well-trained physician leaders have the potential to drive systemic improvements in patient care at a larger scale. By fostering a culture of continuous learning, collaboration, and innovation, physician leaders can influence the implementation of evidence-based practices, quality improvement initiatives, and streamlined care protocols. Effective leadership training empowers physicians to advocate for patient-centered care, promote interdisciplinary collaboration, and implement strategies that prioritize patient safety and positive long-term outcomes. Through effective leadership, physicians can spearhead initiatives that enhance the overall quality, efficiency, and accessibility of trauma surgical services, ultimately benefiting a broader population of patients in need^5^ ^-^ ^7^.

## Challenges and Opportunities in Leadership Training for Trauma Surgeons

While the benefits of leadership training are evident, there exist several challenges that physicians may encounter during their journey to becoming effective leaders in trauma surgery. Time constraints, the demanding nature of the profession, and the inherent pressure of handling critical cases can often hinder the pursuit of leadership development. Moreover, the traditional medical education system may not adequately emphasize the significance of leadership skills, leading to a gap in preparing physicians for the multifaceted responsibilities of a trauma surgeon. However, these challenges present an opportunity for medical institutions and healthcare organizations to prioritize and integrate comprehensive leadership training programs into the curriculum and professional development pathways for trauma surgeons. By investing in structured mentorship, leadership workshops, and experiential learning opportunities, institutions can nurture the next generation of skilled and compassionate physician leaders who are equipped to navigate the complexities of trauma surgery while delivering exceptional patient care^7^ ^-^ ^10^.

In conclusion, leadership training is an indispensable aspect of preparing physicians to excel in the field of trauma surgery. The commitment to developing effective leadership skills is essential for fostering a culture of excellence, collaboration, and continuous improvement within trauma surgical teams. By equipping physicians with the necessary tools and strategies, leadership training not only enhances the quality of care provided to individual patients but also contributes to broader systemic improvements in patient care. While challenges exist, the integration of comprehensive leadership training programs within medical education and professional development pathways presents a promising opportunity to cultivate a new generation of resilient, empathetic, and proficient physician leaders in the field of trauma surgery, ultimately leading to improved patient outcomes and a more robust healthcare system.

## References

[1] Mansfield PR, Neufeld VR (1995). Leadership development in the medical curriculum. Acad Med.

[2] Wong A, Trollope-Kumar K (2014). Reflections an inquiry into medical students' professional identity formation. Med Educ.

[3] Quince T, Abbas M, Murugesu S, Crawley F, Hyde S (2014). Leadership and management in the undergraduate medical curriculum a qualitative study of students' attitudes and opinions at one UK medical school. BMJ Open.

[4] Braun LT, Zottmann JM, Adolf C, Wosnik A, Hahnenkamp K, Harendza S (2016). Leadership training in undergraduate medical education a systematic review. Med Educ Online.

[5] Veronesi G, Kirkpatrick I, Vallascas F (2013). Clinicians on the board what difference does it make?. Soc Sci Med.

[6] Frank JR, Snell LS, Cate OT, Holmboe ES, Carraccio C, Swing SR (2010). Competency- based medical education theory to practice. Med Teach.

[7] Kanter RM (1977). Men and women of the corporation.

[8] Stoller JK, Rose M, Lee R, Dolgan C, Hoogwerf BJ, Montori VM (2009). Applying the four habits model to the hospitalist movement. J Hosp Med.

[9] Zaccaro SJ, Rittman AL, Marks MA (2001). Team leadership. Leadersh Q.

[10] Bryman A (2006). The debate about quantitative and qualitative research a question of method or epistemology?. Br J Sociol.

